# Association between everyday perceived discrimination and cognitive function as mediated by depression in diverse populations: A HABS‐HD Study

**DOI:** 10.1002/alz.089303

**Published:** 2025-01-09

**Authors:** Edna Patricia Mendoza, Emma Nolan, Nicole Phillips, Robert Barber, Sid E. O'Bryant, Zhengyang Zhou

**Affiliations:** ^1^ University of North Texas Health Science Center, Fort Worth, TX, TX USA; ^2^ Vanderbilt University, Nashville, TN USA; ^3^ University of North Texas Health Science Center, Fort Worth, TX USA

## Abstract

**Background:**

Previous research suggests that perceived discrimination is associated with cognitive function impairment, and such association is mediated by depression. With minority populations continuously growing, it is crucial to investigate such relationships in diverse populations. This study aims to examine and compare the above relationships among non‐Hispanic white (NHW), Mexican American (MA), and African American (AA) participants.

**Method:**

A sample size of 1,129 participants (640 AAs, 248 NHWs, 241 MAs) aged 50+ came from the Health and Aging Brain Study – Health Disparities (HABS‐HD). Multiple linear regression was conducted to explore the direct association between perceived discrimination, measured by the Everyday Discrimination Scale mean score, and cognitive function, measured by the Mini Mental State Examination (MMSE) Score. The mediation effect of depression, measured by the Geriatric Depression Scale total score, was evaluated by the indirect effect estimate and confidence interval through Monte Carlo simulations.

**Result:**

Everyday perceived discrimination negatively influenced cognitive function, and the effect was fully mediated by depression across the three populations (estimate for indirect effect = ‐0.16, 95% CI = [‐0.24, ‐0.09]). When stratified, the mediation effect of depression on the association between discrimination and cognitive function remained significant for NHW (estimate for indirect effect = ‐0.37, 95% CI = [‐0.63, ‐0.16]) and MA (estimate for indirect effect = ‐0.42, 95% CI = [‐0.74, ‐0.16]). However, such mediation effect was not observed for the AA population.

**Conclusion:**

Depression mediates the link between everyday discrimination and cognitive decline, but differences between racial/ethnic groups underscore the need for further research into underlying mechanisms among minority groups, including Mexican American and African American populations. Depression interventions may mitigate negative cognitive effects from discrimination. Tailoring such interventions by race/ethnicity and targeting at‐risk groups could optimally promote cognitive health.

